# Randomised Controlled Trial (RCT) of cannabinoid replacement therapy (Nabiximols) for the management of treatment-resistant cannabis dependent patients: a study protocol

**DOI:** 10.1186/s12888-018-1682-2

**Published:** 2018-05-18

**Authors:** Anjali K. Bhardwaj, David J. Allsop, Jan Copeland, Iain S. McGregor, Adrian Dunlop, Marian Shanahan, Raimondo Bruno, Nghi Phung, Mark Montebello, Craig Sadler, Jessica Gugusheff, Melissa Jackson, Jennifer Luksza, Nicholas Lintzeris, Agonist Replacement for Cannabis Dependence (ARCD) study group, Therese Chan, Betty Jago, Lynsey McKendrick, Consuelo Rivas, Ricardo Schwanz, Abigail Yang, Zachary Zavareh, Michelle Hall, Susan Hazelwood, Anthony Winmill, Angelo Barbaro, Kerin Black, Tim Ho, Meryem Jefferies, Jonathon Coreas Jule, Shyam Nagubandi, Mahsa Shahidi, Catherine Silsbury Lisa Snell, Matthew Wijanto

**Affiliations:** 1 Discipline of Addiction Medicine, Central Clinical School, University of Sydney, Sydney, NSW Australia; 2 The Langton Centre, Drug and Alcohol Services, South Eastern Sydney Local Health District, Sydney, NSW Australia; 3 School of Psychology, University of Sydney, Sydney, NSW Australia; 4 National Drug and Alcohol Research Centre, University of New South Wales, Sydney, NSW Australia; 5 Drug and Alcohol Clinical Services, Hunter New England Local Health District, Newcastle, NSW Australia; 6 School of Medicine, University of Tasmania, Hobart, TAS Australia; 7 Centre for Addiction Medicine, Cumberland Hospital, Western Sydney Local Health District, Sydney, NSW Australia

**Keywords:** Cannabis, Cannabis withdrawal, Agonist replacement, Nabiximols, Marijuana, Study protocol

## Abstract

**Background:**

The cannabis extract nabiximols (Sativex®) effectively supresses withdrawal symptoms and cravings in treatment resistant cannabis dependent individuals, who have high relapse rates following conventional withdrawal treatments. This study examines the efficacy, safety and cost-effectiveness of longer-term nabiximols treatment for outpatient cannabis dependent patients who have not responded to previous conventional treatment approaches.

**Methods/Design:**

A phase III multi-site outpatient, randomised, double-blinded, placebo controlled parallel design, comparing a 12-week course of nabiximols to placebo, with follow up at 24 weeks after enrolment.

Four specialist drug and alcohol outpatient clinics in New South Wales, Australia.

One hundred forty-two treatment seeking cannabis dependent adults, with no significant medical, psychiatric or other substance use disorders.

Nabiximols is an oromucosal spray prescribed on a flexible dose regimen to a maximum daily dose of 32 sprays; 8 sprays (total 21.6 mg tetrahydrocannabinol (THC) and 20 mg cannabidiol (CBD)) four times a day, or matching placebo, dispensed weekly. All participants will receive six-sessions of individual cognitive behavioural therapy (CBT) and weekly clinical reviews.

Primary endpoints are use of non-prescribed cannabis (self-reported cannabis use days, urine toxicology), safety measures (adverse events and abuse liability), and cost effectiveness (incremental cost effectiveness in achieving additional Quality Adjusted Life Years). Secondary outcomes include, improvement in physical and mental health parameters, substance use other than cannabis, cognitive functioning and patient satisfaction measures.

**Discussion:**

This is the first outpatient community-based randomised controlled study of nabiximols as an agonist replacement medication for treating cannabis dependence, targeting individuals who have not previously responded to conventional treatment approaches. The study and treatment design is modelled upon an earlier study with this population and more generally on other agonist replacement treatments (e.g. nicotine, opioids).

**Trial registration:**

Australian and New Zealand Clinical Trial Registry: ACTRN12616000103460 (Registered 1st February 2016).

**Electronic supplementary material:**

The online version of this article (10.1186/s12888-018-1682-2) contains supplementary material, which is available to authorized users.

## Background

Approximately 183 million people aged 15-64 years, (3.8%) of the global population currently use cannabis worldwide, dwarfing the use of all other globally regulated substances [1, 2] . In Australia, it is estimated that about 6.6 million people aged 14 years or older have used cannabis at some stage in their lives [3], compared with approximately 22.2 million in the USA [4, 5]. Close to 10.4% of adults in Australia between the age of 14 and over have used cannabis in the past 12 months [6], of which approximately 10% report dependent patterns of use, making it the most common illicit drug of dependence [7].

Cannabis dependence is associated with a range of health problems including cognitive, psychiatric, cardiovascular and respiratory disorders [1, 7, 8], and considerable societal burden [9]. Cannabis ranks second of all illicit drugs in hospital associated costs [10], is the primary drug of concern in 24% of Australian alcohol and other drug treatment services (AODTS) [11], and is identified as a problem drug for 55% of addiction-related treatment episodes [11].

The effectiveness of existing treatments for cannabis use disorders is far from satisfactory but consistent with many other addictive disorders. Reviews of current ‘best practice’ psychosocial interventions (e.g. cognitive behavioural therapy (CBT)) indicate that around 80% of patients relapse within 1-6 months [12]. Treatment of acute cannabis withdrawal is associated with similar relapse rates [13, 14]. More effective approaches are required for people seeking treatment for cannabis use related problems, and as with the treatment of other chronic addiction conditions, there is particular interest in the development of treatment approaches that combine medication with psychosocial interventions [15]. Whilst the importance of medication to support current best practice psychosocial interventions has been identified [15], there are as yet no efficacious pharmacotherapies for cannabis dependence [16]. Medication trials for cannabis dependence are an emerging area of research [17]. Most trials have either been laboratory based or focused only on treating withdrawal symptoms during initial abstinence rather than longer term relapse prevention [18]. Medicines tested for withdrawal treatment – with limited efficacy include antidepressants such as bupropion, [19] the mood stabilizers divalproex [20] and lithium [14], the α_2−_adrenergic agonist lofexidine [21] and the supplement N-acetylcysteine (NAC) [22].

A promising alternative is agonist replacement (or substitution) cannabinoid pharmacotherapies, akin to opioid or nicotine replacement treatment [23]. Studies of cannabinoid agonist medications in withdrawal treatment indicate successful amelioration of withdrawal symptoms. The rationale for agonist medications in cannabis dependence is that they provide a safer route of administration (than smoking), should reduce unsanctioned drug use by preventing withdrawal and reducing cravings [13], and attenuate the acute effects of smoked cannabis [24, 25], potentially facilitating greater engagement in psychosocial interventions. Together, these anticipated effects should provide those also receiving psycho-social interventions and related support to make the necessary lifestyle changes, and distance themselves from drug-related cues, prior to tapering off agonist medication.

Dronabinol, an orally administered synthetic analogue of THC, dose-dependently reduced withdrawal symptoms in the laboratory [20], and improved retention in an outpatient setting [26, 27]. Nabilone, another synthetic THC analogue was efficacious in laboratory experiments, but is as yet untested in clinical settings [21]. A double blind placebo-controlled RCT [13] of nabiximols, an oromucosal spray with approximately equal parts of the cannabinoids THC and CBD, demonstrated successful suppression of withdrawal symptoms and cravings during inpatient detoxification, with greater rates of treatment completion than placebo. However, the high rates of relapse from continuous abstinence following discharge (over two thirds in both groups, but delayed in the nabiximols group) indicate limited longer-term benefits of a 6-day nabiximols regimen.

The high rate of relapse after acute medication-assisted withdrawal highlights the need for longer-term outpatient trials of cannabinoid replacement therapies [15, 16].

Only one RCT has examined longer-term cannabinoid agonist replacement treatment. Levin and colleagues [28], compared a 12-week outpatient course of dronabinol to placebo in 121 cannabis dependent treatment seekers. Although dronabinol was well tolerated, had higher treatment retention and reduced withdrawal symptoms, there was no advantage of dronabinol over placebo in achieving abstinence from illicit cannabis, the primary end-point of the study.

The pharmacological profile of nabiximols may have advantages over other available THC agonist medications. Nabiximols is approved in Australia and a number of countries for symptomatic relief of moderate to severe spasticity in multiple sclerosis. It is an oralmucosal spray containing extracts from *Cannabis sativa* plants grown under licence in the UK by the company GW Pharmaceuticals, containing 2.7 mg THC and 2.5 mg CBD per 0.1 ml spray, with small amounts (4 mg/ml) of other plant-derived cannabinoids. The buccal route of administration provides a rapid onset of action and more favourable pharmacokinetics than oral THC or dronabinol [26], more closely mimicking smoked cannabis use. The anxiolytic effects of CBD present in nabiximols may also ameliorate cravings and anxiety associated with dependent cannabis use and withdrawal.

Two outpatient pilot studies have examined the potential role of nabiximols as an agonist replacement therapy for cannabis withdrawal [29, 30]. In a within-subject placebo controlled randomised controlled trial with 8 participants, high nabiximols doses (up to 108 mg THC/100 mg CBD daily) were well tolerated and significantly reduced cannabis withdrawal symptoms in those achieving abstinence from illicit cannabis use [29]. The same research group have recently reported positive clinical findings (78% abstinence) in an open label 12 week course of treatment with nabiximols (average daily dosage 77.5 mg THC/ 71.7 mg CBD) and psychological treatment in four participants [30]. These studies highlight nabiximols potential as a longer term agonist replacement approach, however, to date, no outpatient large scale randomised controlled trial has examined the safety and efficacy of nabiximols as a substitution medication in the management of cannabis dependence.

### Study objectives

This study examines the efficacy, safety and cost-effectiveness of nabiximols in the outpatient treatment of cannabis dependent treatment seeking patients who have not previously responded to conventional psychosocial interventions. Specific study objectives are:

- Primary Objective 1: To examine the effects of nabiximols vs. placebo on a range of cannabis treatment efficacy outcomes, primarily, changes in illicit cannabis use during treatment and effects on retention in treatment.
- Primary Objective 2: To examine the adverse event profile and abuse liability of nabiximols as a take home treatment for cannabis use disorder.
- Primary Objective 3: To assess the costs and health related quality of life (HRQoL) associated with nabiximols treatment and the potential societal savings (decreased health care, improved productivity, and decreased criminal behaviours).
- Secondary objectives: To examine changes in health related outcomes during outpatient treatment with nabiximols, including a range of mental and physical health dimensions, cognitive function, and psychosocial functioning.

## Methods

### Study design

**Fig. 1:**
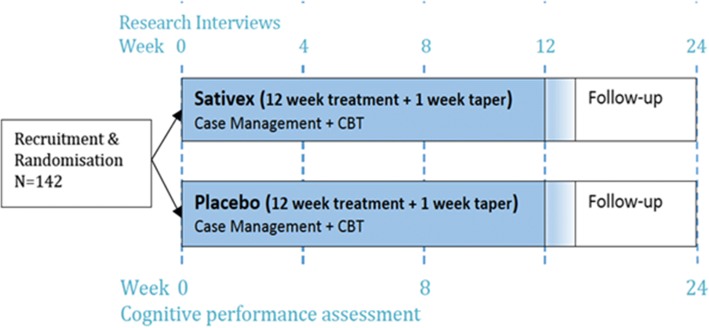
Overview of study design

This project is a phase III multisite (four-sites) outpatient randomised double-blind placebo controlled parallel design comparing a 12-week course of buccally administered nabiximols to placebo (Fig. 1). Both groups receive structured ‘best practice’ individual counselling based on cognitive behavioural therapy principles, regular case management and clinical reviews over the course of the trial. Medications (nabiximols, placebo) are dispensed on a weekly basis, and study medication is discontinued in week 12 using tapering doses. Participants are followed up at week 24, 12 weeks after discontinuation of nabiximols/placebo treatment. All participants are also followed-up for confidential research interviews, (irrespective of completion of the trial intervention) at baseline (week 0), weeks 4, 8, 12 and 24. Overview of study procedures and sequence of events is provided in Table 1.

**Table 1:** Table of schedule of events

	Study period															
	Screen	Enrol	Post-allocation													Follow up
Timepoint*	-t_1_	0	Wk 1	Wk2	Wk3	Wk4	Wk5	Wk6	Wk7	Wk8	Wk9	Wk 10	Wk 11	Wk 12	Wk 13	Wk 24
Enrolment																
Phone screen (Eligibility)	X															
Informed consent for medical screen	X															
Medical screen/assessment (Eligibility)	X															
Informed consent for main study participation		X														
Allocation		X														
Intervention																
Medication *[Nabiximols or placebo]* dispensed			X	X	X	X	X	X	X	X	X	X	X	X	X	
*Nursing clinical reviews*			X	X	X	X	X	X	X	X	X	X	X	X	X	
*Medical clinical reviews*			X	X		X				X				X		
*CBT sessions*				X		X		X		X		X		X		
*Urine drug screen*		X	X	X	X	X	X	X	X	X	X	X	X	X		X
Assessments																
Research Interviews. Variables include: Cannabis & other substance use (TLFB), CWS, AEs, Aberrant medication behaviour (mod ORBIT), SF-6D (QOL), WHO Health and Performance Questionnaire: CT version, SF-36 (Physical and Mental health), DASS-21, PHQ-15, OTI: BPI, Crime, Satisfaction, Test blind (week 12 only)		X				X				X				X		X
Clinical (Nursing/medical) Review variables: AEs, Aberrant medication behaviour (weigh bottles), ratings dose adequacy, ATOP & BPRS at 4 week intervals, reason for study termination Wk 24.			X	X	X	X	X	X	X	X	X	X	X	X		
Cognitive assessment. Variables include Blood samples (pre/post cognitive testing), Cognitive testing, Abuse liability (subjective liking, strength, Physiological response)		X				X										X

### Regulatory and funding

The design complies with requirements of the Consolidated Standards of Reporting Trials (CONSORT) guideline [31, 32] (see Additional file 1 for SPIRIT CONSORT checklist), and has received ethical clearance from the Human Research Ethics Committee of South East Sydney Local Health District (HREC/14/POWH/701), with relevant local site specific approvals. The University of Sydney is the study sponsor. A National Health and Medical Research Council (NHMRC) of Australia Project Grant (#1088902) supports the research costs of the study. Health service costs are predominately supported by the participating NSW Health services. Study medications (nabiximols, placebo) are provided free to the study by GW Pharmaceuticals. The study is registered on the Australian New Zealand Clinical Trials Registry (ACTRN12616000103460).

A Data Safety Monitoring Board (DSMB) comprising of an independent statistician, a researcher with cannabinoid expertise, and an addiction medicine specialist with extensive expertise in clinical trials oversees the study.

### Sites

Four clinical sites across three Local Health Districts in New South Wales, Australia were selected to host the study based on their location, history of providing specialist treatment services for cannabis dependent users, and capacity to participate and co-ordinate clinical research activities. Two clinical sites are based in the South East Sydney Local Health District (The Langton Centre and St George Hospital), one in Hunter New England Local Health District (Newcastle Community Health Services) and one in Western Sydney Local Health District (Centre for Addiction Medicine).

### Participants

#### Sample size

Based on the primary outcome measure (efficacy, safety and cost-effectiveness of nabiximols in outpatient treatment of cannabis dependence), a total sample size of 142 participants (71 per group) is required to achieve around 22% abstinence rates at 12-weeks for the placebo group (ascertained by average of previous drug administered clinical trials) and approximately 44% abstinence rates for the nabiximols group (based on clinical judgement of double the rate of abstinence than placebo) with 80% power (two-tailed) and α = 0.05.

#### Eligibility criteria

Inclusion criteria are: (a) age 18 to 65 years; (b) meet ICD-10 cannabis dependence criteria; (c) inability to stop cannabis use in previous attempts, operationalised as relapse to cannabis use within one month of a substantive cessation attempt (including prior episodes of counselling, medication, or withdrawal treatment aimed at abstaining from cannabis use), and (d) being willing and able to provide informed consent and follow study procedures, including agreeing to not drive or operate heavy machinery and females of child bearing potential agree to use reliable contraception during the duration of the trial.

#### Exclusion criteria

Exclusion criteria for the study are: (a) presence of another substance use disorder other than nicotine or caffeine (i.e. alcohol, other illicit or prescription drug dependence, including methadone or buprenorphine treatment for opioid dependence); (b) severe medical (e.g. severe chronic pain, severe hepatic, cardiovascular or renal impairment) or psychiatric disorder (e.g. unstable schizophrenia, recent drug-induced psychosis, severe mood disorder), assessed at medical screen; (c) women who are pregnant, lactating or planning to become pregnant; (d) concerns regarding safe storage of medication; (e) not available for follow up (e.g. expected travel or incarceration); (f) mandated by court to attend treatment and maintain abstinence for a substance use disorder; (g) history of epilepsy or recurrent seizures; and (i) current active or recent (within past month) treatment for cannabis use disorder.

#### Early termination criteria

Early termination criteria for the study include: (i) severe adverse side effects or deteriorating physical or mental health; (ii) violation of treatment centre rules and conditions (e.g. violence towards staff or other patients); (iii) non-compliance with trial protocol, including missing/not returning medication for more than two consecutive weeks or persistent refusal to participate in trial procedures (such as, non-adherence with study medications, counselling, clinical reviews, case management urine drug screen, bloods, research interviews or monitoring).

### Recruitment

The study is promoted via; (i) referrals from Drug & Alcohol treatment services; (ii) media advertising, including local newspapers and postcard placements; and (iii) online advertising through University of Sydney website.

A three-step recruitment procedure is used for the study. Interested individuals contact either the central trial coordinator or site-specific researchers by telephone, and are screened for potential eligibility. Information collected at telephone screen relate to the selection criteria, including demographics; recent cannabis and other substance use in past 28 days, Cannabis Severity of Dependence Scale (SDS) score [33]; participation in treatment for substance use disorder in last 28 days; self-reported severe mental and physical health conditions; prior quit attempts; willingness and ability to participate in the study, including weekly clinic attendance. If deemed broadly eligible at telephone screen, the potential participant is invited to attend a detailed medical assessment with a study medical officer at one of the four sites, generally within 1-2 weeks of the telephone screen.

The medical assessment is conducted by a study medical officer (SMO) - an Addiction Medicine Specialist or delegated senior addiction medicine trainee, and involves a comprehensive clinical assessment examining patient goals, substance use and treatment history, physical and mental health, and psychosocial conditions. In addition to the physical and mental state examinations, clinical investigations, such as, urine drug screen for cannabis and other substance use, urine β-hCG in women to exclude pregnancy, and blood tests are carried out as clinically indicated (e.g. Liver Function Tests if concerned regarding hepatic function). The SMO completes the eligibility checklist following the assessment, and if the patient is eligible and interested in participating in the study, they are referred to the study researcher at each site.

The researcher then provides detailed verbal and written study information, and obtains written informed consent to participate in the study. Upon signing the informed consent form, participants are enrolled into the study.

### Randomisation and blinding

An independent statistician created a randomisation schedule, which was then given to the lead trial pharmacist at each site. Participants are blindly randomised in a 1:1 ratio between groups, using the variable 8-block randomisation schedule of 80 codes per site to maintain blinding. Stratification occurs at each of the four sites (aiming for a 1:1 random allocation at each site). Randomisation and group allocation happen after enrolment (informed consent) and prior to Day 1 of the study, enabling study medications to be prepared by the study pharmacist at each site. The randomisation schedule and nabiximols and placebo canisters are only identifiable to trial pharmacists at each site, who themselves have no contact with participants. All participants, clinicians and researchers involved in service delivery, data collection and analysis remain blinded to group allocation (active or placebo) until the end of the study.

### Clinical intervention

#### Study medications

Nabiximols is an oromucosal spray delivered through a mechanically actuated pump, with each spray delivering 100 μL (2.7 mg THC and 2.5 mg CBD). The placebo medication consists of an alcohol base and peppermint oil flavouring present in the active nabiximols medication, but does not include any cannabinoids and plant-based terpenoids. A previous study [13] demonstrated the ability to maintain blind dosing of nabiximols versus placebo in cannabis dependent patients in an inpatient setting. All dispensed medication canisters are labelled with the subjects’ name, trial site, week number, medication dose, expiry date and prescribing doctors’ name.

The proposed dosing regimen for nabiximols is based upon experience from previous studies with cannabis dependent patients [13, 29, 30], and the principles of dose titration used in similar clinical paradigms (opioid agonist replacement therapy). Nabiximols and placebo doses are individually titrated by the SMO with each participant to optimise clinical effect (reduce cravings and withdrawal) and safety (avoidance of side effects, such as intoxication). Doses are reviewed and titrated by the clinician and the patient regularly (daily for first two days of Week 1, then at weekly intervals). Day 1 dose is two sprays (5.4 mg THC, 5.0 mg CBD) four times a day (QID), days 2 and 3 maximum dose is four sprays (10.8 mg THC, 10 mg CBD) QID. Thereafter, the maximum daily doses during the ‘maintenance’ period (to end week 12) are 32 sprays (86.4 mg THC, 80 mg CBD) per day divided into four doses (21.6 mg THC, 20 mg CBD QID). These nabiximols doses are considerably greater than those conventionally used for treating other indications (e.g. a daily maximum dose for multiple sclerosis is 32.4 mg THC, 30 mg CBD), reflecting the high THC tolerance of cannabis dependent individuals and the need for high doses to effectively ameliorate withdrawal symptoms and cravings. During week 13, participants have the option of tapered reductions of their study medication at approximately 10-15% per day, in order to minimise any medication discontinuation effects (e.g. withdrawal).

#### Clinical reviews

Weekly clinical reviews with a nurse and/or SMO include: a Time Line Follow Back (TLFB) [34] of their use of study medication, cannabis, nicotine, alcohol and other drugs in the past week since last clinical review; a review of any adverse events; separate participant and clinician assessment of medication dose adequacy (using a 5-point Likert scale e.g. 1 = ‘much too low’, 3 = ‘about right’ to 5 = ‘much too high’); participant rating of cannabis withdrawal severity using Cannabis Withdrawal Scale (CWS) [35], general physical and mental health and psychosocial conditions, examination (including blood pressure (BP), pulse rate (PR), evidence of intoxication or withdrawal, or other findings of note) and the opportunity to address other issues identified by the participant. In addition to weekly reviews conducted by nursing staff (baseline, days 1 and 2, weeks 3, 5, 6, 7, 9, 10, and 11), the SMO reviews the participant at weeks 2, 4, 8 and 12, at which time they also formally assess any adverse events reported since the last medical review and complete the Australian Treatment Outcomes Profile (ATOP) [36], a validated clinician-completed instrument that includes participant ratings of physical and mental health and range of clinical risks, such as, child protection, violence, homelessness) and the Brief Psychiatric Rating Scale (BPRS) [37] to formally assesses psychiatric symptoms that may occur with high dose THC use [38]. The SMO assess whether the participant is globally deteriorating and warrants study discontinuation. See Table 1 for schedule of events.

#### Psychosocial intervention

In conjunction with receiving medication treatment, participants are provided with a minimum of six structured counselling sessions based on cognitive behavioural therapy (CBT) [39]. The CBT sessions use a range of strategies and interventions, such as, understanding cannabis and the patient, preparing the patient for change and various strategies, ways to manage withdrawal and relapse prevention. Participation in the counselling program is encouraged, and all participants must attend at least 2 sessions (an initial assessment and one further follow up) in order to continue in the study. Participants may negotiate additional counselling sessions with the therapist beyond the 12-week intervention period. Counsellors complete de-identified research Clinical Record Forms (CRFs) after each session to identify themes or areas covered according to the CBT intervention as a strategy to record adherence with the counselling intervention.

### Outcome and treatment process measures

Outcome measures for each of the study objectives are described below, and summarised in Tables 1 and 2.

**Table 2:** Table of all clinical and research measures included in study

Domain	Name	Description of measure	How and when administered
Basic	Demographics	10 Questions: basic demographics including; age, gender, employment, relationship status, education level, living situation and Aboriginal and Torres Strait Islander background.	Research Interview: Week 0 (baseline) day 1
Cannabis Use	Cannabis Use	4 semi-structured questions: exploring frequency, amount and method of cannabis use in the last 4-weeks	Research Interview: Weeks 0, 4, 8, 12, 24
	Cannabis Withdrawal Scale (CWS) [35]	19 items on a 10-point Likert Scale, with 0 = Not at all to 10 = Extremely. The CWS is used as a diagnostic instrument in clinical and research settings to assess cannabis withdrawal symptoms. Participants are asked how they feel over the last 24 h in 8 domains; irritability, depression, anxiety, cannabis cravings, physical symptoms, sleep difficulty, restlessness and appetite.	Research Interview and Clinical Reviews: Weekly
	Marijuana Craving Questionnaire-short form (MCQ) [66]	12 items on a 7-point Likert scale, with 1 = strongly disagree to 7 = strongly agree. The MCQ is a self-report instrument that assesses marijuana craving along 4 dimensions; compulsivity, emotionality, expectancy and purposefulness.	Research Interview: Weeks 0, 4, 8, 12, 24
	Cannabis Problems Questionnaire (Adult revised- CPQ-R) [67]	27 items on a 11-point Likert scale, with 0 = doesn’t apply to me to 10 = strongly apply to me. The CPQ-R measures acute and physical consequences, psychological consequences, and social consequences of cannabis use.	Research Interview: Weeks 0, 4, 8, 12, 24
	Self-coping and efficacy for quitting cannabis questionnaire [68]	20 items on a 7-point Likert scale, with 1 = not at all confident to 7 = very confident. Participants are asked to indicated their confidence in their ability to resist the temptation to use cannabis in a variety of interpersonal and intrapersonal situations.	Research Interview: Weeks 0, 4, 8, 12, 24
	Modified Time Line Follow Back *(adapted)* [40]	Using a modified Time Line Follow Back approach examining cannabis use in preceding 4 week period.	Research Interview: Weeks 0, 4, 8, 12, 24
Other Substance Use	Australian Treatment Outcome Profile (ATOP) [36]	3 Part Questionnaire:	Research Interview: Weeks 0, 4, 8, 12, 24
		1. Substance use; items refer to substance use over the last 4-weeks, in particular: alcohol, amphetamines, benzodiazepines, heroin, other opioids, cocaine, other substances and tobacco.	
		2. Injecting risk behaviour; 2-items exploring injecting behaviour in the last 4-weeks.	
		3.Health and Well-being; 11-items investigating the participants’ accommodation arrangements, psychological and physical health and quality of life.	
	FagerstrÖm Test for nicotine dependence (FTND) [69]	6 item questionnaire that measures nicotine dependency, with a total available score of 10.	Research Interview: Weeks 0, 4, 8, 12, 24
	The Alcohol Use Disorders Identification Test (AUDIT) [70]	10 item questionnaire covering domains of alcohol consumption, drinking behaviour and alcohol related problems. Responses to each question are scored from 0 to 4, giving a maximum possible score of 40.	Research Interview: Weeks 0, 4, 8, 12, 24
	Opioid Related Behaviours in Treatment (ORBIT) scale *(modified)* [71]	8 item questionnaire on a 5-point Likert scale ranging from 0 = never to 4 = very often. The questionnaire explores opioid-related behaviours that are divergent, unexpected, non-adherence to medication, unsanctioned diversion of medication to others, hazardous use or misuse. ORBIT questions modified for cannabis dependent population.	Research Interview: Week 12
Mental Health	Depression, Anxiety and Stress scale (DASS) Short-form [51]	21 item questionnaire on a 4-point Likert scale, ranging from 0 = did not apply to me at all to 3 = applied to me very much or most pf the time. The DASS 21 measures the severity of symptoms of depression, anxiety and stress and enables clinicians to measure a patient’s response to treatment.	Research Interview: Weeks 0, 4, 8, 12, 24
	Primary Care Post Traumatic Stress Disorder (PC-PTSD) [72]	4 item questionnaire that explores whether a patient has ever experienced a traumatic event (e.g. a serious accident, physical/sexual assault/abuse, natural or political disaster etc.) over the course of their life. If so, participants answer a series of Yes/No questions.	Research Interview: Weeks 0, 4, 8, 12, 24
	Brief Psychiatric Rating Scale (BPRS) [37]	18 item questionnaire on a 7-point Likert scale, ranging from 1 = not present to 7 = extremely severe. Clinicians measure psychiatric symptoms such as depression, anxiety, hallucinations and unusual behaviour.	Research Interview: Weeks 0, 4, 8, 12, 24
Physical Health	Short form 36 health survey questionnaire (SF-36) [50]	36 item questionnaire using a mixed scale. Domains covered in the survey include; physical functioning, role limitations due to physical problems, social functioning, bodily pain, general mental health, role limitations due to emotional problems, vitality and general health problems.	Research Interview: Weeks 0, 4, 8, 12, 24
	Brief Pain Inventory - Short form (BPI) [53]	5 items taken from Brief Pain Inventory-Short form using a 10-point Likert scale, ranging from 0-no pain to 10 = worst pain imaginable. Participants are asked to think about any abnormal pain experienced in the last 7 days and rate the intensity and interference of the pain in the participants’ life.	Research Interview: Weeks 0, 4, 8, 12, 24
	Athens Insomnia Scale (AIS) [54]	7 item questionnaire using a 4-point Likert scale ranging from 0 = none to 4 = very severe. The AIS is a self-assessment psychometric instrument designed for quantifying sleep difficulty based on the ICD-10 criteria.	Research Interview: Weeks 0, 4, 8, 12, 24
	Sheehan Disability Scale (SDS) [73]	3 item instrument using a 10-point Likert scale ranging from 0 = not at all to 10 = very severe. The SDS asks participants to best rate their impairment with work, social life/leisure activities and family life/home responsibilities.	Research Interview: Weeks 0, 4, 8, 12, 24
	Visual Analogue Scale of client ratings on medication effects [42]	5 item questionnaire using a scale from 0 to 100 measuring subjective liking and strength of drug effect, comparability to cannabis, sedation, bad effects and subjective physiological effects	Research Interview: Weeks 0, 4, 8, 12, 24
Social/ Other	Opioid Treatment Index- Crime (OTI-Crime) [55]	8 questions modified from the full Opioid Treatment Index. The modified version concentrates on questions relating to criminality and focuses on the frequency of recent criminal behaviour in four areas: property crime, drug dealing, fraud and crimes involving violence.	Research Interview: Weeks 0, 4, 8, 12, 24
	Health and Work Performance Questionnaire (HPQ) -Short Form Absenteeism and Presenteeism [74]	4 Questions modified from the Health and Work Performance questionnaire (HPQ). The HPQ is designed to estimate the workplace costs of health problems in terms of reduced job performance, sickness absence, and work-related accidents/injuries.	Research Interview: Weeks 0, 4, 8, 12, 24
	National Evaluation of Pharmacotherapies for Opioid Dependence (NEPOD) - Health Service Utilisation (HSU) [75]	5 questions adapted from the NEPOD study. The HSU section of the NEPOD focusses on health services used in the last 4-weeks, frequency of use and cost of service to the participant.	Research Interview: Weeks 0, 4, 8, 12, 24
	Testing the Blind	5 questions exploring the participants’ subjective opinion of study drug allocation.	Research Interview: Weeks 4, 8, 12, 24
	Patient’s Global Impression of Change Scale (PGICS) [64]	1 question modified from the Global Rating of Change (GLOC) survey exploring the participants’ subjective opinion of change since starting study.	Day: 85
Cognitive Assessment	Wechsler Test of Adult Reading (WTAR) [61]	Verbal pronunciation of 50 words with irregular phoneme-to-grampheme conversion. Total correct recorded	Research Interview: Weeks 0
	Eriksen Arrow Flankers (with no-go) [56]	Speed of response to target stimuli flanked with either neutral, congruent or incongruent stimuli (24 each). Random 10% (*n* = 8) trials require with-holding of response (no-go). Reaction time; errors and false alarms recorded	Research Interview: Weeks 0, 4, 24
	1,2,3-Back Task [58]	Identification of repetition in a rapid sequence of stimuli, either 1, 2, or 3 stimuli prior (30, 75, 75 stimuli respectively; 25% targets). Reaction time, accuracy, false-positives recorded	Research Interview: Weeks 0, 4, 24
	Symbol-Digit Substitution Task (SDMT) [59]	Decoding symbols related to presented digit; 87 trials; Reaction Time and Accuracy recorded	Research Interview: Weeks 0, 4, 24
	Stop-Signal Task (SST) [57]	Withholding of already initiated motor response after a delay; 12 target trials of 48; reaction time, errors, stop signal response time recorded	Research Interview: Weeks 0, 4, 24
	Rapid Visual Information Processing (RVIP) [60]	Response to high working memory load (3 back) stimuli; 24 targets in 300 stimuli; reaction time; accuracy and false positive responses recorded	Research Interview: Weeks 0, 4, 24
	Rey Auditory Verbal Learning Task [62]	Retention of a 15 item word list presented on 5 occasions. Number of words recalled on each presentation, following presentation of a novel list of 15 words; and then after brief and long delays recorded. Recognition of target stimuli also assessed.	Research Interview: Weeks 0, 4, 24

#### Objective 1

To examine cannabis treatment efficacy outcomes, including changes in illicit cannabis use during treatment and treatment retention.

- Illicit cannabis use is quantified as self-reported number of days of illicit cannabis use and average daily amount of cannabis use in grams at the 4 weekly research interviews using modified Timeline Followback [40] techniques. The primary end-point is self-reported illicit cannabis use days during the maintenance phase of treatment (weeks 1-12). Objective measures of illicit cannabis use will be determined from weekly urine collection with quantitative analysis of urinary THC, THC-COOH, CBD and other cannabinoids. 4-weekly point prevalence abstinence during maintenance phase (weeks 1-4, 5-8, 9-12), and post-treatment period (week 24) will be ascertained by combining self-report data from researcher interviews and urinalysis.
- Treatment retention (days in protocol treatment) are recorded from clinical records. Participants who do not attend for more than two consecutive weeks are deemed to have dropped out of treatment, and the last scheduled day of dosing is calculated as the end of treatment date.

#### Objective 2

To examine the adverse event profile, and the abuse liability of nabiximols as a take home treatment for cannabis use disorder.

- Adverse events are assessed and addressed during clinical assessments with the study medical officer at 4-weekly appointments. At the end of study participation, the SMO records the severity of each AE (mild, moderate, severe), the outcome (ongoing or resolved, with or without treatment), and attribution to study medication.
- Abuse liability: Adherence to study medication is estimated by participants returning their medication canisters at the weekly clinical review and weighing the amounts of medication used (equivalent to a pill count). Aberrant medication behaviours (missed doses, extra ‘unsanctioned’ doses, misuse or diversion) are assessed by self-report at researcher interviews using the modified ORBIT [41], a validated aberrant medication behaviours self-report instrument. In addition, a series of subjective assessments of abuse liability using the Visual Analogue Scale (VAS) [42], (0-100) of subjective liking, comparability to cannabis, strength of effect and subjective physiological effects are conducted 4-weekly at researcher interviews.

#### Objective 3

To assess the costs and health related quality of life (HRQoL) associated with the provision of nabiximols for treatment of resistant cannabis use disorder and the potential societal savings (decreased health care, improved productivity, and changes in criminal behaviors).

- Cost effectiveness: Consistent with other drug treatment cost effectiveness evaluations [43–45], the primary outcome is Quality Adjusted Life Years (QALY) measured by the SF-6D [46], (at 4 weekly research interviews) determined using area under the curve analysis [47] for each individual. Resources included are all clinical services provided as trial interventions (see Treatment Process Measures below), adverse event management, self-reported health care utilization outside of the trial (hospital, emergency department, primary care and other specialist health services) as well as self-reported participation in criminal activity that will be costed using unit costs (CPI adjusted if necessary) [48]. Lost productivity and personal costs are collected by structured self-report (WHO Health and Performance Questionnaire: Clinical Trials Version) [49]. The costs will be summed and combined with the outcome measure, and the incremental cost-effectiveness ratio [ICER = (C_Nabiximols_-C_Control_)/(E_Nabiximols_-E_Control_)] calculated.

#### Secondary objectives

To examine changes in health-related outcomes during outpatient treatment with nabiximols, including mental and physical health, cognitive performance, and psychosocial functioning

- Other substance use (alcohol, opioids, stimulants, benzodiazepines, tobacco) are recorded by self-report number of days used in the past 28 days using the ATOP at 4-week research interview.
- Health outcomes and psychosocial function**.** The SF-36 [50] is administered at 4-week research interviews to assess dimensions of physical and mental health and psychosocial function. Mental health will be also be assessed using the Depression, Anxiety and Stress Scale (DASS-21) [51] at 4-weekly research interviews and the BPRS at 4-weekly medical reviews. Patient reported ratings of physical health are also assessed using the Physical Health Questionnaire-15 (PHQ-15) [52]. Pain severity and pain interference is assessed using the Brief Pain Inventory [53]. Subjective sleep ratings are assessed using the Insomnia Sleep Inventory [54]. Self-reported drug related crime (e.g. drug dealing, income generating crime) are examined using the Crime Section of the Opiate Treatment Index [55].
- Cognitive function is assessed by the researcher at baseline (week 0, day 1), during the maintenance phase (week 4), and at follow-up (week 24). A targeted series of computerised tests sensitive to acute THC effects are conducted, specifically an acute battery: Eriksen Flanker Task [56], Stop Signal Task [57], N-Back [58], Digit-Symbol Substitution [59], and Rapid Visual Information Processing [60] as well as a control measure (Wechsler Test of Adult Reading [61]) and a measure of memory and learning (Ray Auditory Verbal Learning Test, RAVLT [62]). At week 4 assessments, cognitive testing (acute battery) are performed 30 min prior to (trough) and 45-60 min after (peak effects) supervised dosing. Blood samples at trough and peak are taken for plasma cannabinoid levels (THC, 11-OH THC, THC-COOH, CBD, 11-OH CBD) to assist in the interpretation of findings. The acute battery uses computerised tests within the Penscreen system [63] that create random numbers for stimuli to minimise learning effects. Similarly, repeated memory assessment use parallel versions of the RAVLT. It will be of particular interest to examine whether nabiximols is associated with cognitive improvement relative to Placebo and relative to baseline. Week 24 cognitive performance assessment is examined to investigate within-subject longitudinal changes over time.
- Patient reported outcome and satisfaction measures: Many of the study outcomes use ‘patient reported outcome measures’ of specific domains. The Patient Global Impression of Change Scale (PGICS) [64] examines the participant’s assessment of change in their global condition at week 12 since entering treatment in the trial. Participants are also asked questions relating to their satisfaction of treatment medication and dose and their likelihood of recommending it to others.

#### Treatment ‘process’ measures

details regarding participation in trial interventions are collected on paper clinical record forms (CRF) and include details regarding doses of trial medication used (reported at weekly clinical reviews), participation in medical, counselling and clinical review sessions as well as reasons for study termination (completed protocol, treatment drop-out, medical discharge, administrative discharge, incarceration). At the completion of the ‘maintenance’ medication phase of the trial (week 12 researcher interview), participants are asked to estimate which medication group they were assigned as a means of testing the blind.

### Research procedures

#### Research interviews

The research assistant at each site conducts research interviews at weeks 0 (baseline), 4, 8, 12 and week 24. Details of the instruments and timing of administration are shown in Table 2.

#### Urinalysis and blood pathology testing

Blood and urine samples are collected in accordance with the National Statement on Ethical Conduct in Human Research (2007). All blood and urine samples are taken and stored de-identified using the patients study ID code.

Urine drug screens are collected during the medical screen and on day 1 of commencing the trial to confirm cannabis use and check for other drug use other than cannabis (prior to study medication being administered). Subsequent urine samples are collected weekly at the clinical review (and at week 24 research interview), and stored at − 20 °C until the end of the trial, with results not being available to the clinical or research staff until the completion of all data collection. The urine samples will enable cannabinoid metabolite profiles to be charted through time, as a means to identify ongoing illicit cannabis use, using creatinine corrections [65].

Blood samples are taken from all participants at baseline, week 4 (immediately before and after cognitive testing) and week 24 to determine serum cannabinoid (THC, 11-OH THC, THC-COOH, CBD, 7-OH CBD) levels. 10mls of blood are collected at each time-point and immediately centrifuged at 4 °C for 15 min at 1500 RPM and plasma pipetted into 4 × 1 ml aliquots, immediately frozen at − 20 °C. A Shimadzu 8030 triple quadrupole mass spectrometer will be used to analyse serum cannabinoids (LCMS; Shimadzu Corp, Kyoto, Japan).

## Statistical analyses

Chi square and ANOVA tests will be performed to identify any baseline covariates that differ between groups. Prior to carrying out primary and secondary analysis, the percentage of missing values in the raw dataset and Little’s MCAR test will be used to determine if multiple imputation of missing values is required (except for missing urine where cannabis use will be assumed to have taken place). The multiple imputation will be done if the percentage of missing values exceeds 5%.

All analyses will use Intention-to-treat, which is defined as any person who is randomised to one of the study arms and receives at least one dose of study medications. Mixed Models for Repeated Measures (MMRM) will compare groups on changes in outcome variables (cannabis use and secondary outcomes) in the medication phase with the multiply imputed dataset, (assuming that Little’s MCAR at Random test confirms the data to be missing at random). In addition, we will perform a sensitivity analysis based on only those with complete data, and compare results to that from the MI dataset analysis.

Adverse Events will be analysed using chi-square. A Cox proportional hazards model will compare retention in treatment between study arms, controlling for potential confounds. The impact of the intervention on post-medication outcomes will compare changes in cannabis use outcomes at baseline and at follow up between groups using MMRMs.

Family-wise error corrections will control for Type 1 errors where multiple comparisons are performed within a particular analysis where post hoc contrasts are performed to further explore interesting (significant) findings.

## Study limitations

This study has a number of possible limitations. One issue relates to retention in study protocol. Participation in the study is voluntary, and study procedures span 24 weeks, including 13 weeks of study treatment interventions. The lengthy treatment duration in the study is generally longer than conventional treatment of cannabis dependence, and participants may be deterred from entering the trial or prematurely terminate study participation, which in turn may have implications in assessing effectiveness of treatment outcomes and research follow-up. Similarly, individuals enrolling in a medication study may have different levels of interest or commitment to the CBT in the study, which may enhance heterogeneity of outcomes.

The study uses a flexible dosing approach to medication. Whilst a fixed regimen – or indeed a structured comparison of different doses (e.g. high or low) is often used in many medication studies – experience from opioid agonist clinical and research practice highlights the need to tailor doses to individual requirements, reflecting differing physiological tolerance, adverse event profiles and behavioural characteristics of patients. Nevertheless, the different doses used may increase heterogeneity of outcomes and limit the ability to detect significant differences if there is indeed a major dose effect.

Measures of illicit cannabis use during treatment are the primary study outcome, with self-reported use (via the TLFB) as the primary endpoint. Participants may have a response bias and report inaccurate drug use to both clinicians and researchers. Whilst objective assessment of illicit cannabis use using urine drug screening should enhance self-report validity, it remains to be seen whether illicit cannabis use can be differentiated from prescribed nabiximols use. Various toxicological approaches are being examined to validate the identification of illicit cannabis use in nabiximols prescribed patients (to be described elsewhere).

## Conclusion

There is a need for more effective treatment approaches for cannabis dependent patients who are unable to discontinue their illicit use through psychosocial interventions alone. Longer-term agonist replacement treatment approaches rather than acute withdrawal management are likely to be more effective, with the combination of THC:CBD nabiximols preparation being potentially advantageous over synthetic THC analogues. This is the first large-scale outpatient RCT of nabiximols for this population, and has required the development of clinical and research methods specific to agonist treatment with a plant-derived cannabinoid formulation, building upon clinical research models previously used in opioid agonist treatment approaches.

## Additional file

Additional file 1: CONSORT 2010 checklist of information to include when reporting a randomised trial. (PDF 69 kb)

## Abbreviations

ATOP: Australian Treatment Outcomes Profile
BPI: Brief Pain Inventory
BPRS: Brief Psychiatric Rating Scale
CBD: Cannabidiol
CBT: Cognitive behavioural therapy
CONSORT: Consolidated Standards of Reporting Trials
CRF: Clinical record form
CWS: Cannabis Withdrawal Scale
DASS-21: Depression, Anxiety and Stress Scale-21
DSMB: Data Safety Monitoring Board
HRQoL: Health related quality of life
ISI: Insomnia Sleep Inventory
NHMRC: National Health and Medical Research Council
ORBIT: Opioid Treatment Index- Crime
PGICS: Patient Global Impression of Change Scale
PHQ-15: Physical Health Questionnaire-15
QALY: Quality Adjusted Life Years
QID: Four times a day
RAVLT: Ray Auditory Verbal Learning Test
RCT: Randomised controlled trial
SF-36: Short form 36 health survey questionnaire
SMO: Study medical officer
THC: Tetrahydrocannabinol
TLFB: Timeline Follow Back
VAS: Visual Analogue Scale
